# Development of an HPLC–MS/MS method for the determination of ceftolozane/tazobactam in bronchoalveolar lavage fluid

**DOI:** 10.4155/fsoa-2018-0079

**Published:** 2018-11-15

**Authors:** Christina A Sutherland, Can Ozbal, David P Nicolau

**Affiliations:** 1 Center for Anti-Infective Research & Development Hartford Hospital, Hartford, CT, 06102 USA; 2 PureHoney Technologies, Inc., Billerica, MA, 01821 USA; 3 Division of Infectious Diseases, Hartford Hospital, Hartford, CT, 06102 USA

**Keywords:** BAL, ceftolozane, HPLC–MS, tazobactam

## Abstract

**Aim:**

We describe the validation of an HPLC–MS/MS method to analyze ceftolozane and tazobactam simultaneously in saline matrixes.

**Materials & methods:**

An Agilent 1260 HPLC interfaced to an Agilent 6470 triple-quadrupole mass spectrometer was used for quantification. A reverse-phase column running a gradient of water and acetonitrile containing 0.1% formic acid mobile phase at a flow rate of 1.0 ml/min provided chromatographic fractionation. Tazobactam^15^N_3_ was used as the internal standard. The standard curves were linear over a range of 0.02–0.5 μg/ml.

**Conclusion:**

This methodology represents a simple, reproducible approach to the determination of drug concentrations with accuracy and precision for pharmacokinetic studies undertaken with this recently US FDA-approved antimicrobial therapy.

## Lay abstract

We describe the validation of an assay to analyze ceftolozane and tazobactam simultaneously in saline matrixes. This methodology represents a simple, reproducible approach to the determination of drug concentrations. This method can be modified to determine human concentrations of this antibiotic, which would be important for doctors to determine appropriate patient dosing.

Ceftolozane/tazobactam (ZERBAXA^®^, Merck & Company, Inc., NJ, USA) is a combination product consisting of a cephalosporin-class antibacterial drug and a β-lactamase inhibitor formulated in a two-to-one ratio. This antibacterial was approved by the US FDA in 2014. Ceftolozane/tazobactam is indicated for use in adults with complicated intra-abdominal infections when used with metronidazole and complicated urinary tract infections [1]. Ceftolozane/tazobactam has been found to be an active agent against *Pseudomonas aeruginosa* including drug-resistant strains from both intensive care unit and nonintensive care unit settings [2]. As a result of ceftolozane/tazobactam use in pulmonary infections the determination of concentrations in biological fluids of the lung will be increasingly important.

Currently published methods reporting the assay of ceftolozane/tazobactam use HPLC, and to date no articles describe the assay in detail with the use of MS [3,4]. The purpose of this report is to describe a simple, reproducible and selective HPLC–MS/MS method for the use in bronchoalveolar lavage fluid (BAL).

## Experimental

### Instrumentation & reagents

Quantitative analysis was performed using an Agilent 6470 HPLC, interfaced to an Agilent 6470 triple-quadrupole mass spectrometer with an Agilent Jet Stream source in electrospray ionization (ESI) mode (Agilent Technologies, CA, USA). The samples were placed in an Agilent G1367A well plate autosampler and were chilled to 4°C. A 40 μl aliquot of sample was injected on a Zorbax Eclipse C18 4.6 × 100 mm, 3.5 μm column (Agilent Technologies). The mobile phase consisted of HPLC-grade water with 0.1% formic acid as solvent A and acetonitrile with 0.1% formic acid as solvent B. An Infinity II binary pump (Agilent Technologies) was used for 4 min linear gradient of solvent B from 5 to 80% followed by a 1 min washout with 100% B and 2.5 min re-equilibration of the column at 5% B using a flow rate of 1 ml/min. The total run time was 8 min per sample. Mass spectrometric analysis was performed using positive ESI in multiple reaction-monitoring mode with both Q1 and Q3 resolution set to unit. For ceftolozane 667.2–199 transition was used while 304.2–168.3 and 301.3–168.3 transitions were used for tazobactam^15^N_3_ and tazobactam, respectively. The fragmentor voltage was set to 135 V and the collision energy was set to 15 V for all three analytes. The ESI parameters are as follows: gas temp = 275°C; gas flow = 12 l/min; nebulizer = 35 psi; sheath gas temp = 300°C; sheath gas flow = 12 l/min; capillary = 3000 V; nozzle voltage = 1000 V.

Ceftolozane and tazobactam standard powders were provided by Merck and Company, Inc. Stable isotope tazobactam^15^N_3_ (Toronto Research Chemicals, Toronto, Canada) was used as the internal standard. HPLC grade acetonitrile, HPLC grade water and LC–MS grade trifluoroacetic acid used were purchased from Fisher Scientific (NH, USA). The formic acid is from Acros Organics (Thermo Fisher Scientific, NJ, USA).

### Standard solutions & controls

Ceftolozane and tazobactam were dissolved in water as recommended, and diluted the 2000 μg/ml stock standard. Tazobactam ^15^N_3_ stock solution of 0.5 μg/ml was prepared in water according to the manufacturer's recommendations and 0.06 ml of the stock internal standard solution was added to 1 ml of sample for a final concentration of 0.03 μg/ml in all standards and samples. The volume of the internal standard stock solution was adjusted to provide a 0.03 μg/ml solution in samples to be analyzed since volume was a limiting factor in some samples. Saline (0.9% sodium chloride for injection; Hospira, Inc., IL, USA) was be used as the matrix for any BAL samples. Saline was chosen as the surrogate matrix since the BAL fluid is mostly made up of saline due to the instillation of saline during the procedure. Collecting blank BAL fluid on patients or animals is not always practical. Nine-point calibration curves with 0, 0.2, 0.4, 0.6, 0.8, 1, 2, 3 and 5 μg/ml of both ceftolozane and tazobactam were prepared. Ceftolozane and tazobactam were combined together to form one set of standard sample tubes to mimic the unknown samples. Low-, mid- and high-qualifiers used in the experiment were prepared at 0.3, 0.9 and 4 μg/ml. A standard curve and qualifiers were diluted in 0.9 ml of saline for a final concentration of 0, 0.02, 0.03, 0.04, 0.06, 0.08, 0.09, 0.1, 0.2, 0.3, 0.4 and 0.5 and run with each batch of samples. A total of 60 samples can be analyzed in 8 h. Blank saline and saline with internal standard (0 μg/ml) were assayed with each standard curve. Aliquots of the standards and internal standard were stored at -80°C until analysis.

Tazobactam^15^N_3_ was chosen for the internal standard for both ceftolozane and tazobactam assays since there is no stable isotope available for ceftolozane at this time. A tazobactam ester was tried for the internal standard for the tazobactam, which produced inconsistent results. Isotopes for the ceftolozane compound could be produced and will need to be tested for future assays.

## Materials & methods

### Sample preparation

A 100 μl sample of standard, quality control or unknown sample along with a 60 μl aliquot of internal standard was diluted into 0.9 ml of saline. The sample was vortexed for 10 s and pipetted into a single micro centrifuge tube. No further sample preparation was needed since the BAL fluid is mostly saline and the samples were further diluted in saline. All samples and standards were frozen at -80°C for shipment on dry ice to PureHoney Technologies, Inc. (MA, USA) for HPLC–MS analysis. Prior to analysis the samples were transferred to a 4°C fridge for 1 h to thaw. Any additional thawing was performed at room temperature as necessary.

The HPLC–MS data were analyzed using Agilent MassHunter. Curve fitting for the standard curves was performed using SigmaPlot 12.0 software using a 1/× weighting.

### Assay validation

Calibration curves were generated by using the ratio from the ceftolozane and tazobactam peak to that of the internal standard. A weighted (1/concentration) least square regression analysis was used to generate the linear regression equation for compound. The zero standard (saline sample processed with internal standard) was not used in generating the linear regression equation. This linear regression equation was used to calculate the concentrations of the quality controls and unknown samples. Linearity of the standard curve was assessed with the correlation coefficient by comparing the normalized ratios of ceftolozane and tazobactam with internal standard against the theoretical concentrations.

A calibration curve consisted of a zero sample and eight standard samples. Three quality controls with low, middle and high concentrations were used to evaluate the precision and accuracy. Precision was determined by taking the SD divided by the average. Accuracy was determined by taking the calculated concentration divided by the theoretical concentration. The lower limit of quantification (LLOQ) for the assay was evaluated on five samples.

Room-temperature studies of ceftolozane/tazobactam were conducted in triplicate on each quality control sample at 22°C. Quality control samples of 0.3, 0.9 and 4 μg/ml were thawed and kept at room temperature for the length of time that would be required for regular sample processing. The freeze and thaw stability was performed by completely thawing the quality controls at room temperature and refreezing at -80°C for 24 h. The freeze–thaw cycle was done onceand then analyzed on the second cycle.

## Results

### Linearity, precision & accuracy

Linearity was demonstrated with the correlation coefficient (r) for each calibration curve of ≥0.996 for ceftolozane and tazobactam standard curves (n = 7). The ceftolozane slope was 31.47 ± 19.29 (mean ± SD), and the intercept was 0.08 ± 0.04 (mean ± SD). The tazobactam slope was 16.13 ± 3.58 (mean ± SD) and the intercept was -0.02 ± 0.02 (mean ± SD).

The summary data for the inter- and intra-day precision and accuracy in both the ceftolozane and tazobactam saline are shown in Tables 1 & 2, respectively.

**Table 1.** **Precision and accuracy of ceftolozane in saline. **

**Parameters**	**Ceftolozane concentration (μg/ml)**		

**Low (0.03)**	**Medium (0.09)**	**High (0.4)**	
Inter-run (n = 7)			

Mean	0.03	0.09	0.40

SD	0.002	0.005	0.009

Precision	6.69%	6.26%	2.19%

Accuracy	97.75%	95.77%	99.83%

Intra-run (n = 10)			

Mean	0.03	0.08	0.40

SD	0.001	0.003	0.023

Precision	4.57%	4.18%	5.79%

Accuracy	98.45%	91.33%	100.19%

SD: Standard deviation.

**Table 2.** **Precision and accuracy of tazobactam in saline. **

**Parameters**	**Tazobactam concentration (μg/ml)**		

**Low (0.03)**	**Medium (0.09)**	**High (0.4)**	
Inter-run (n = 7)			

Mean	0.03	0.10	0.41

SD	0.001	0.003	0.009

Precision	2.66%	2.64%	2.26%

Accuracy	103.37%	105.68%	102.55%

Intra-run (n = 10)			

Mean	0.03	0.09	0.41

SD	0.002	0.003	0.020

Precision	5.75%	3.69%	4.92%

Accuracy	98.17%	103.63%	102.13%

SD: Standard deviation.

### LLOQ

**Figure 1.**
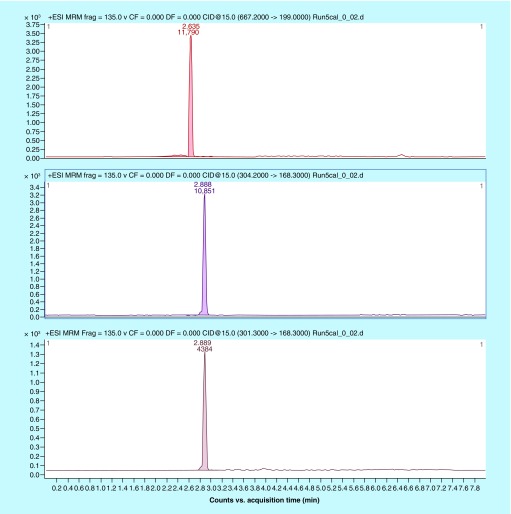
**Lower limit of quantification of 0.02 μg/ml.** Ceftolozane (top trace), tazobactam internal standard (middle trace) and tazobactam (bottom trace).

The LLOQ of 0.02 μg/ml (Figure 1) for ceftolozane and tazobactam was chosen as the concentration for the lowest standard sample. The precision and accuracy of LLOQ (n = 5) for ceftolozane were 2.30 and 112.9%, respectively. The precision and accuracy of LLOQ (n = 5) for tazobactam were 3.15 and 99.8%, respectively.

### Stability

Ceftolozane and tazobactam saline quality controls were stable at a room temperature of approximately 22°C for 2 h with <6% degradation. Ceftolozane/tazobactam quality control samples were stable for two freeze–thaw cycles with an average of ≤10% degradation. Ceftolozane/tazobactam quality control samples were not stable for three freeze–thaw cycles (≥10% degradation).

The long-term stability samples had <9% degradation after 48 days at -80°C, which was previously determined by HPLC methods [5]. Stability at -20°C and stability of swine BAL samples were not tested.

### Recovery

Recovery experiments were performed in triplicate for each of the quality control concentrations. Values of percent recoveries of ceftolozane/tazobactam were calculated by comparing the calculated concentration of a sample with the internal standard in saline representing 100% recovery to that of a BAL sample. The ceftolozane recovery of the 0.03, 0.09 and 4 μg/ml samples was 100.7% ± 2.94%, 103.4% ± 1.27% and 104.9% ± 1.12%, respectively. The tazobactam recovery of the 0.03, 0.09 and 4 μg/ml samples was 105.9% ± 2.80%, 104.8% ± 3.70% and 101.1% ± 6.77%, respectively.

### Sample results

A pharmacodynamic study was undertaken in infected swine to study the BAL concentrations of ceftolozane/tazobactam. Samples were taken during the BAL procedure and analyzed for drug concentrations. The publication with results of that study is pending [6]. The concentrations ranged from below detectable limit (<0.02 μg/ml) in samples obtained prior to dosing to 9.99 μg/ml for ceftolozane and 1.63 μg/ml for tazobactam seen at the peak time point. We did not see any interference in samples taken prior to dosing. All samples taken after the prior time point were run with a saline dilution to bring them within the range of the standard curve (0.02–0.5 μg/ml).

## Discussion

As a result of increasing antimicrobial resistance and the need for optimal pharmacodynamic exposures in the critically ill population, knowledge of drug concentrations within the bronchopulmonary space is increasingly needed. We developed an efficient, reliable and sensitive HPLC–MS/MS method to assess the BAL fluid concentrations of ceftolozane/tazobactam, a new antibacterial compound being utilized in the management of pulmonary infections. The lower detection limit compared with conventional HPLC can aid in detecting concentrations in both highly diluted samples (i.e., BAL fluid) as well as low-volume samples such as that of interstitial fluid collected during *in vivo* microdialysis procedures. The precision and accuracy of this method are well within the acceptable limits of 15% of the actual value and 20% at LLOQ as defined by FDA guidance [7]. This method was successfully used for the analysis of swine BAL samples from a pharmacokinetic study.

The traditional HPLC assay for ceftolozane/tazobactam, used to assay plasma samples, takes 26 min per sample. Many of the autosamplers only hold up to 48 samples. With eight standards per run, only 40 samples can be analyzed per day using HPLC methods. The HPLC–MS assay for ceftolozane/tazobactam totaled 8 min per sample.

## Conclusion & future perspective

This assay successfully determined the BAL concentrations of infected swine dosed with ceftolozane/tazobactam. While human BAL samples should be similar to that of swine BAL, the assay may need to be further modified. Other matrixes (i.e., plasma) could be developed for the HPLC–MS assay as a time-saving method for laboratories. Over the next 5–10 years, advances in equipment could lead to more selective and faster antibiotic assays. Advances in experimental designs aimed at lowering the sample volume required for assays could help determine pediatric as well as limited-volume body fluid samples.

**Executive summary**

- A ceftolozane/tazobactam assay was validated using an HPLC–MS/MS method for the use in bronchoalveolar lavage fluid.
- The assay is simple, reproducible and selective for ceftolozane/tazobactam in saline over the range of 0.2–5 μg/ml.
- This assay was successfully used to determine the bronchoalveolar lavage concentrations of infected swine dosed with ceftolozane/tazobactam.
